# Clinical and Economic Outcomes Associated With Use of Liposomal Bupivacaine Versus Standard of Care for Management of Postsurgical Pain in Pediatric Patients Undergoing Spine Surgery

**DOI:** 10.36469/jheor.2021.21967

**Published:** 2021-04-14

**Authors:** Robert Tracy Ballock, John Seif, Ryan Goodwin, Jennifer H. Lin, Jessica Cirillo

**Affiliations:** 1 Department of Orthopedic Surgery, Cleveland Clinic, Cleveland, OH; 2 Department of Pediatric Anesthesiology, Cleveland Clinic, Cleveland, OH; 3 Pacira BioSciences, Inc., Parsippany, NJ

**Keywords:** spinal fusion, health economics, opioids, costs of hospital care, hospitalized child, adolescent medicine, anesthesiology, pediatrics

## Abstract

**Background:** Approximately 60% of hospitalized children undergoing surgery experience at least 1 day of moderate-to-severe pain after surgery. Pain following spine surgery may affect opioid exposure, length of stay (LOS), and costs in hospitalized pediatric patients. This is a retrospective cohort analysis of pediatric patients undergoing inpatient primary spine surgery.

**Objectives:** To examine the association of opioid-related and economic outcomes with postsurgical liposomal bupivacaine (LB) or non-LB analgesia in pediatric patients who received spine surgery.

**Methods:** Premier Healthcare Database records (January 2015–September 2019) for patients aged 1–17 years undergoing inpatient primary spine surgery were retrospectively analyzed. Outcomes included in-hospital postsurgical opioid consumption (morphine milligram equivalents [MMEs]), opioid-related adverse events (ORAEs), LOS (days), and total hospital costs. A generalized linear model adjusting for baseline characteristics was used.

**Results:** Among 10 189 pediatric patients, the LB cohort (n=373) consumed significantly fewer postsurgical opioids than the non-LB cohort (n=9816; adjusted MME ratio, 0.53 [95% confidence interval (CI), 0.45–0.61]; P<0.0001). LOS was significantly shorter in the LB versus non-LB cohort (adjusted rate ratio, 0.86 [95% CI, 0.80–0.94]; P=0.0003). Hospital costs were significantly lower in the LB versus non-LB cohort overall (adjusted rate ratio, 0.92 [95% CI, 0.86–0.99]; P=0.0227) mostly because of decreased LOS and central supply costs. ORAEs were not significantly different between groups (adjusted rate ratio, 0.84 [95% CI, 0.65–1.08]; P=0.1791).

**Discussion:** LB analgesia was associated with shorter LOS and lower hospital costs compared with non-LB analgesia in pediatric patients undergoing spine surgery. The LB cohort had lower adjusted room and board and central supply costs than the non-LB cohort. These data suggest that treatment with LB might reduce hospital LOS and subsequently health-care costs, and additional cost savings outside the hospital room may factor into overall health-care cost savings. LB may reduce pain and the need for supplemental postsurgical opioids, thus reducing pain and opioid-associated expenses while improving patient satisfaction with postsurgical care.

**Conclusions:** Pediatric patients undergoing spine surgery who received LB had significantly reduced in-hospital postsurgical opioid consumption, LOS, and hospital costs compared with those who did not.

## INTRODUCTION

Recent improvements in the assessment and management of pediatric pain include the Joint Commission on Accreditation of Health Care Organizations 2001 mandate to treat and assess pain in all patients and increased inclusion of pediatric patients in drug development studies.^1^ However, despite these improvements, 60% of hospitalized children undergoing surgery experience at least 1 day of moderate-to-severe pain after surgery.^2^ In particular, pediatric patients undergoing spine surgery are likely to experience postsurgical pain, which may in turn affect exposure to opioids, length of stay (LOS) in the hospital, and medical costs.^3,4^

Despite the associated opioid-related adverse events (ORAEs), opioids are commonly prescribed for pain management following pediatric surgery.^5–9^ Prescription of opioids can be associated with misuse and development of persistent opioid use, particularly in adolescents, and ORAEs that may lead to substantial health-care resource utilization.^5,10^ From 1997 to 2012, the incidence of hospitalizations for prescription opioid poisonings increased nearly 2-fold among children and adolescents aged 1–19 years, with the highest overall incidence reported in adolescents (aged 15–19 years).^11^ Multimodal analgesic regimens, which include nonopioid regional anesthetics, are being implemented by hospitals to not only better manage postsurgical pain and improve recovery, but also to minimize in-hospital postsurgical opioid consumption.^12^

Liposomal bupivacaine (LB) is a long-acting single-dose infiltration multivesicular liposome formulation of the local anesthetic bupivacaine that provides prolonged bupivacaine release over several days.^13^ Adult patients treated with LB demonstrated reduced postsurgical pain and opioid consumption when administered LB via local infiltration or as an interscalene branchial plexus nerve block in multiple surgical procedures.^13^ However, use of LB to treat postsurgical pain in orthopedic surgery has rarely been assessed in pediatric patients. Data are also lacking regarding the effectiveness of LB on hospital cost of care in these patients. This retrospective analysis aimed to comprehensively assess both opioid-related and economic outcomes, including opioid consumption, ORAEs, hospital LOS, and total hospital costs, associated with the use of LB or non-LB analgesia in pediatric patients undergoing primary spine surgery.

## MATERIALS AND METHODS

### Study Design

We performed a retrospective analysis of the deidentified Premier Healthcare Database, which contains administrative data since January 2000 from >1000 US hospitals and health-care systems, including academic and nonacademic institutions in both urban and rural locations.^14^ Over 231 million unique patients representing ~25% of US inpatient admissions are included in the database. The database contains information on hospital characteristics, patient visit characteristics, specialties of admitting and attending physicians, health-care payers, and patient information such as demographics, disease states, costs for billed services, and patient discharge health status obtained from standard hospital discharge billing. This analysis was exempt from institutional review board review requirements per Department of Health and Human Services policy (Title 45 Code of Federal Regulations, Part 46 of the United States) because patient records were deidentified.

### Patient Population

**Figure 1.**
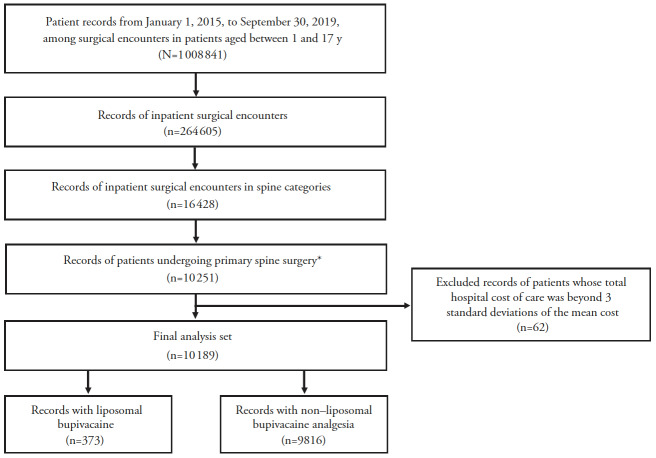
Surgical Records Retrieval Workflow for Pediatric Patients Undergoing Spine Surgery Process for retrieving surgical records from the Premier Healthcare Database for patients aged 1–17 years receiving liposomal bupivacaine or non–liposomal bupivacaine analgesia after primary spine surgery. *Includes discectomy, lumbosacral fusion, other fusion, laminectomy, or other spine surgery.

Data were analyzed from pediatric patients aged 1–17 years who underwent inpatient primary spine surgery including discectomy, lumbosacral fusion, other fusion, laminectomy, or other spine surgery, which were identified by International Classification of Diseases, Ninth Revision (ICD-9) and ICD-10 codes (Supplemental Table 1) between January 1, 2015, and September 30, 2019, and received either LB analgesia as identified by the Premier Healthcare Database standard charge master codes (Supplemental Table 2) or who did not receive LB analgesia for pain management following the procedure (Figure 1). Patients were excluded if they had multiple records of the same primary surgery admission or if their hospital costs were ≥3 standard deviations beyond the mean cost because they were considered outliers. The follow-up time for the patients covered the entire hospitalization period from admission to discharge.

### Study Variables

The primary treatment exposure variable in this study was the use of LB as a binary variable, generated by the Premier Healthcare Database standard charge master codes. The primary outcome of interest was inpatient opioid prescription, which was extracted from standard charge master codes for opioids and was converted into total morphine milligram equivalents (MMEs). Additionally, LOS in the hospital, reported in days, and total cost of hospital care, in US dollars, were extracted from the Premier Healthcare Database hospital encounter summary. The other secondary outcomes of interest were ORAEs, including cardiovascular, central nervous system, gastrointestinal, respiratory, skin, and other complications, defined by ICD-9 and ICD-10 diagnosis codes, using a previously published approach.^15^

The patient demographic information comprised age (in years) at hospital admission, sex, race (White, Black, other), Quan-Charlson Comorbidity index defined by ICD-9 and ICD-10 diagnosis codes and recorded at hospital admission prior to surgery, patient-controlled analgesia (yes, no), surgery type (discectomy, lumbosacral fusion, other fusion, laminectomy, other spine surgery), and surgical year (2015–2019). The hospital characteristics included location (urban, rural), hospital size (0–299, ≥300 beds), hospital teaching status (yes, no), and geographic region (northwest, southwest, south, west).

### Statistical Analyses

Continuous variables were summarized using the mean and standard deviation, and categorical variables were summarized using the frequency and percentage. A generalized linear model was used to compare LB versus non-LB analgesia in relation to clinical (opioid consumption during hospital stay in MMEs [conversion factors for morphine equivalents are provided in Supplemental Table 3] and ORAEs) and economic (LOS and total hospital costs) outcomes for all inpatient spine surgery types combined, assuming gamma distribution with log link function for MME and cost outcomes, negative binomial distribution with log link function for LOS, and binomial distribution with logit link function for ORAEs. To control for patient and hospital characteristics, the analytic model was adjusted for age, sex, race, Quan-Charlson Comorbidity Index, location, teaching hospital status, hospital size (bed number), geographic region, use of patient-controlled analgesia, and surgery type. All statistical analyses were performed using SAS software, version 9.4. A *P* value of <0.05 was considered statistically significant.

When the modeling distribution for total MME intake was tested using the family test with ordinary least squares method, the gamma distribution appeared to be valid with an observed gamma of 2.14 (95% confidence interval [CI], 2.08–2.19). Although the test for total cost of care seemed to suggest Poisson distribution (observed gamma of 1.02 [95% CI, 0.94–1.11]), we observed very similar results to our original analysis when Poisson distribution was applied. For the ORAEs outcome, logit link appeared to be appropriate with the goodness of link assessment (observed parameter, 0.03 [95% CI, <0.001–1.18]).

## RESULTS

### Patient Characteristics

Of >2 million patients screened, 10 189 pediatric patients were identified for this analysis, with 373 patients in the LB cohort and 9816 in the non-LB cohort (Figure 1). Generally, baseline characteristics were comparable between the LB and non-LB cohorts, although the LB cohort appeared to be older than the non-LB cohort (mean age, 14 and 12 years, respectively; Table 1).

**Table 1.** Patient and Hospital Characteristics

	**LB (n=373)**	**Non-LB (n=9816)**	***P* value**
Age, mean (SD), y	14.2 (2.6)	12.0 (4.5)	<0.0001
Sex, n (%)			0.1280
Female	235 (63.0)	5797 (59.1)	
Male	138 (37.0)	4019 (40.9)	
Race, n (%)			0.8668
Black	55 (14.7)	1368 (13.9)	
White	248 (66.5)	6648 (67.7)	
Other	70 (18.8)	1800 (18.3)	
Quan-Charlson Comorbidity Index, mean (SD)	0.2 (0.7)	0.3 (0.9)	<0.0001
0–1, n (%)	344 (92.2)	8078 (82.3)	
>1, n (%)	29 (7.8)	1738 (17.7)	
Index surgery year, n (%)			<0.0001
2015	60 (16.1)	2136 (21.8)	
2016	100 (26.8)	2507 (25.5)	
2017	79 (21.2)	2276 (23.2)	
2018	55 (14.7)	1645 (16.8)	
2019	79 (21.2)	1252 (12.7)	
PCA, n (%)			0.0003
Yes	82 (22.0)	1479 (15.1)	
No	291 (78.0)	8837 (84.9)	
Teaching hospital, n (%)			0.0072
Yes	288 (77.2)	6948 (70.8)	
No	85 (22.8)	2868 (29.2)	
Location, n (%)			0.0005
Rural	8 (2.1)	652 (6.6)	
Urban	365 (97.9)	9164 (93.4)	
Provider region, n (%)			<0.0001
Northwest	34 (9.1)	1553 (15.8)	
Southwest	150 (40.2)	1591 (16.2)	
South	162 (43.4)	5405 (55.1)	
West	27 (7.2)	1267 (12.9)	
Bed size, n (%)			<0.0001
000–299	173 (46.4)	1988 (20.2)	
≥300	200 (53.6)	7828 (79.8)	
Spine surgery type			<0.0001
Discectomy	6 (1.6)	167 (1.7)	
Fusion (lumbosacral)	42 (11.3)	571 (5.8)	
Fusion (other)	253 (67.8)	5012 (51.1)	
Laminectomy	4 (1.1)	168 (1.7)	
Other	68 (18.2)	3898 (39.7)	

Abbreviations: LB, liposomal bupivacaine; PCA, patient-controlled analgesia; SD, standard deviation.

Approximately 60% of patients in each treatment cohort were female, and approximately two-thirds of patients in each treatment cohort were White; the mean Quan-Charlson Comorbidity Index was 0.2 and 0.3 for the LB and non-LB cohorts, respectively. Less than 1% of patients (n=98) were identified as receiving epidural analgesia. Baseline hospital characteristics and year of surgery were mostly similar for both treatment cohorts. Spinal fusion was the most common surgery in both the LB (79%) and non-LB (57%) cohorts.

### Clinical Outcomes

**Figure 2.**
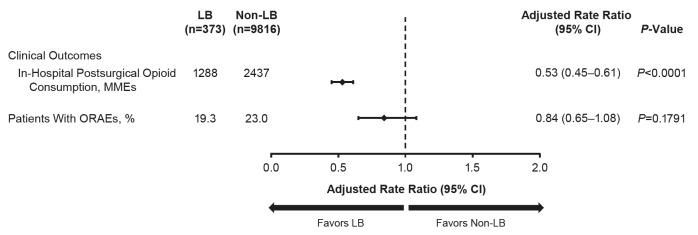
Adjusted Clinical Outcomes After Primary Spine Surgery in Pediatric Patients Model adjusted for age, sex, race, Quan-Charlson Comorbidity Index, location, teaching hospital status, hospital size (bed number), geographic region, patient-controlled analgesia, and surgery type. Abbreviations: CI, confidence interval; LB, liposomal bupivacaine; MME, morphine milli- gram equivalent; ORAE, opioid-related adverse event.

Overall, the LB cohort consumed 47% fewer in-hospital postsurgical opioids than the non-LB cohort (adjusted MMEs, 1288 vs 2437; *P*<0.0001) (Figure 2). Across all procedures, the proportion of patients who experienced ORAEs was not significantly different between the LB and non-LB cohort (adjusted proportion, 19% vs 23%; *P*=0.1791). Unadjusted values are provided in Supplemental Tables 4 and 5.

### Economic Outcomes

**Figure 3.**
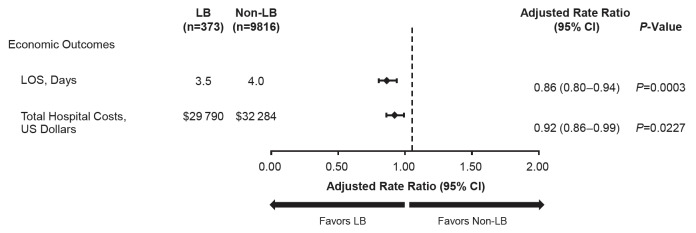
Adjusted Economic Outcomes After Primary Spine Surgery in Pediatric Patients Model adjusted for age, sex, race, Quan-Charlson Comorbidity Index, location, teaching hospital status, hospital size (bed number), geographic region, patient-controlled analgesia, and surgery type. Abbreviations: CI, confidence interval; LB, liposomal bupivacaine; LOS, length of stay.

Overall, the LB cohort demonstrated a 14% shorter LOS compared with the non-LB cohort (adjusted LOS, 3.5 vs 4.0 days; *P*=0.0003) (Figure 3). Patients receiving LB analgesia had significantly lower total hospital costs than patients receiving non-LB analgesia, corresponding to 8% lower total costs with LB (adjusted total cost, $29 790 vs $32 284; *P*=0.0227). The cost savings with LB versus non-LB analgesia were mostly attributable to hospital stay cost by room and board (ie, adjusted hospital stay cost, $6312 vs $7395; 15% savings) and central supply cost (ie, adjusted equipment cost, $8267 vs $9370; 12% savings). Unadjusted values are provided in Supplemental Table 6.

## DISCUSSION

In this retrospective database analysis, pediatric patients with low mean Quan-Charlson Comorbidity Index^16^ undergoing inpatient primary spine surgery who received LB analgesia after surgery had statistically significant lower in-hospital opioid consumption, shorter hospital LOS, and lower total hospital costs compared with those who received non-LB analgesia. These clinical and economic benefits are in accordance with and expand upon results from other retrospective studies examining the use of opioid consumption and LOS for pediatric patients receiving LB or non-LB analgesia for postsurgical pain after spine deformity surgery and LOS for adult Medicare patients undergoing total knee arthroplasty.^17,18^

Adolescents (defined by the World Health Organization as those aged 10–19 years)^19^ may be at increased risk of opioid misuse compared with adults. This may be explained by changes in brain reward and habit formation centers during this developmental period.^5,20^ Exposure to prescription opioids can also contribute to potential opioid misuse and development of persistent opioid use in adolescents. Diversion of prescription opioids in the form of selling, trading, and loaning is common in adolescents, playing a critical role in opioid misuse.^21^ A retrospective analysis of postsurgical opioid refills found that ~5% of opioid-naive adolescents and young adults who filled an opioid prescription after surgery continued to fill their prescription >90 days after surgery; risk factors for persistent use included older age, female sex, and presurgical chronic pain.^5^ Similarly, another retrospective analysis found that 74% (N=346 251) of opioid-naive patients undergoing surgery aged 13 to 21 years filled an initial surgery-related opioid prescription. In the same study, patients who had a family member with long-term opioid use filled an opioid prescription 91 to 180 days after the procedure at a higher rate than those who did not.^6^ Given these previous reports of adolescent persistent opioid use, findings from the current study suggest that use of LB for postsurgical pain management may offer clinical benefits because it was associated with reduced in-hospital postsurgical opioid consumption after spine surgery. Together, use of LB may be beneficial in multimodal pain management strategies aiming to reduce postsurgical opioid exposure and risk for potential opioid misuse and dependence, especially in young patient populations.

In the current study, patients who received LB experienced shorter in-hospital LOS than those who received non-LB analgesia. This finding is consistent with previous reports in adult^22,23^ and pediatric^17^ patients undergoing spine surgery, as well as studies in adult patients in other surgical areas.^24,25^ Use of opioids in the postanesthesia care unit may contribute to ORAEs, possibly resulting in an increase in LOS and total costs.^26,27^ In the current study, lower opioid consumption along with numerically fewer ORAEs might have contributed to the shorter LOS seen in the LB group, as indicated by Olbrecht and colleagues.^4^ In addition, shorter LOS with LB has previously been hypothesized to be associated with earlier mobilization and activity, potentially due to increased pain control, in adult patients after lumbar spine surgery or total hip arthroplasty.^23,25^ However, because time to ambulation was not assessed, its contribution to reduced LOS in the current study cannot be determined.

A previous report has shown that intensive care unit and inpatient room costs contributed greatly to overall hospital costs following idiopathic scoliosis correction surgery in adolescent patients.^28^ In the current study, LB was associated with shorter LOS and lower hospital costs compared with non-LB analgesia in pediatric patients undergoing spine surgery. Because the LB cohort had lower adjusted room and board costs than the non-LB cohort, these data suggest that use of treatments, such as LB, might reduce hospital LOS and subsequently health-care costs. Moreover, the LB cohort had lower adjusted costs for central supply than the non-LB cohort, suggesting that additional cost savings outside the hospital room may also factor into overall health-care cost savings. Cost of complications contributed by postsurgical opioid use and patient recovery experience are critical in drafting guidelines for cost-effective postsurgical patient care.^29^ LB potentially can reduce pain and the need for supplemental postsurgical opioids, thereby reducing pain and opioid-associated expenses, as well as improving patient satisfaction with postsurgical care.

## LIMITATIONS

There are several limitations to consider when interpreting the present findings. One limitation is the potential underreporting of LB use in the database, thus limiting the generalizability of these findings. Fewer than 1% of patients in the entire study cohort were identified as receiving epidural analgesia for postsurgical pain, which is likely an underestimation due to challenges in finding this information among bundled payments in the database, making it difficult to control for non-LB analgesics that were used. The Premier Healthcare Database also does not provide information about perisurgical pathways, which may be heterogenous across institutions; as such, use of LB in spine surgery may reflect implementation of comprehensive enhanced recovery pathways in pediatric and adult patients that may have contributed to the reduction in opioid consumption and shorter hospital stays observed in the present study.^17,30^ Further, as the Premier Healthcare Database is an administrative database lacking clinical details, surgical information related to management protocols and practices was not available. We also did not have data on baseline MME intake, and thus, we were unable to control for this confounding factor in our model. Although the retrospective observational design of this analysis cannot adjust for all confounding variables, this analysis does provide real-world insight into clinical and economic outcomes that are otherwise challenging to assess across randomized controlled trials. Also, we cannot rule out the possibility that the differences we observed could be explained by confounding factors that are not available in the Premier Healthcare Database, including preexisting conditions, prior health-care use, or laboratory results. In addition, this study included several different spine procedures, which could contribute to the heterogeneity of the findings, although we have accounted for surgery types in the regression model. Finally, we were unable to identify whether specific postsurgical pain management protocols were used in the hospitals, which potentially could have influenced the amount of medications used as well as the multimodal approaches included.

## CONCLUSION

In summary, pediatric patients undergoing inpatient primary spine surgery who received LB analgesia experienced improved outcomes compared with patients who did not. The LB cohort showed significant reductions of in-hospital postsurgical opioid consumption, LOS, and costs compared with those who received non-LB analgesia with no significant difference in ORAEs between cohorts. This retrospective observational analysis provides support for the use of LB as part of a multimodal analgesia regimen to manage postsurgical pain and mitigate clinical and economic outcomes in pediatric patients undergoing spine surgery. Future studies confirming these findings in larger sample sizes and in other pediatric surgical procedures are warranted.

### Author Contributions

- Conception and design: Jennifer H. Lin and Jessica Cirillo; (II) Administrative support: Jennifer H. Lin; (III) Provision of study materials or patients: Jennifer H. Lin and Jessica Cirillo; (IV) Collection and assembly of data: Jennifer H. Lin; (V) Data analysis and interpretation: All authors; (VI) Manuscript writing: All authors; (VII) Final approval of manuscript: All authors.

### Conflicts of Interest

RTB is a paid consultant for Pacira BioSciences, Inc. and has received research funding from Pacira BioSciences, Inc. JS is a consultant for Pacira BioSciences, Inc. RG is a consultant for Stryker Spine and Orthopediatrics. JHL and JC are employees of and may hold stock options in Pacira BioSciences, Inc.

### Ethical disclosure

This analysis was independently performed by Pacira BioSciences, Inc. using data from the Premier Healthcare Database. Because this analysis included only deidentified medical records, this study was considered exempt from institutional review board requirements per Department of Health and Human Services policy (Title 45 Code of Federal Regulations, Part 46 of the United States).

## Supplementary Material

Supplemental Table 1. Surgical Procedure Codes

Supplementary Material

## References

[ref-51135] Brislin Robert P., Rose John B. (2005). Pediatric acute pain management. Anesthesiology Clinics of North America.

[ref-51136] Kozlowski Lori J., Kost-Byerly Sabine, Colantuoni Elizabeth, Thompson Carol B., Vasquenza Kelly J., Rothman Sharon K., Billett Carol, White Elizabeth D., Yaster Myron, Monitto Constance L. (2014). Pain prevalence, intensity, assessment, and management in a hospitalized pediatric population. Pain Management Nursing.

[ref-51137] Sheffer Benjamin W., Kelly Derek M., Rhodes Leslie N., Sawyer Jeffrey R. (2017). Perioperative pain management in pediatric spine surgery. Orthopedic Clinics of North America.

[ref-51138] Olbrecht Vanessa A., Ding Lili, Spruance Kristie, Hossain Monir, Sadhasivam Senthilkumar, Chidambaran Vidya (2018). Intravenous acetaminophen reduces length of stay via mediation of postoperative opioid consumption after posterior spinal fusion in a pediatric cohort. Clin J Pain.

[ref-51139] Harbaugh Calista M., Lee Jay S., Hu Hsou Mei, McCabe Sean Esteban, Voepel-Lewis Terri, Englesbe Michael J., Brummett Chad M., Waljee Jennifer F. (2018). Persistent opioid use among pediatric patients after surgery. Pediatrics.

[ref-51141] Harbaugh Calista M., Lee Jay S., Chua Kao-Ping, Kenney Brooke, Iwashyna Theodore John, Englesbe Michael J., Brummett Chad M., Bohnert Amy S., Waljee Jennifer F. (2019). Association between long-term opioid use in family members and persistent opioid use after surgery among adolescents and young adults. JAMA Surgery.

[ref-51142] Anderson K. Tinsley, Bartz-Kurycki Marisa A., Ferguson Dalya M., Kawaguchi Akemi L., Austin Mary T., Kao Lillian S., Lally Kevin P., Tsao KuoJen (2018). Too much of a bad thing: Discharge opioid prescriptions in pediatric appendectomy patients. Journal of Pediatric Surgery.

[ref-51143] Tepolt Frances A., Bido Jennifer, Burgess Stephanie, Micheli Lyle J., Kocher Mininder S. (2018). Opioid overprescription after knee arthroscopy and related surgery in adolescents and young adults. Arthroscopy: The Journal of Arthroscopic & Related Surgery.

[ref-51144] Horton Joshua Dean, Munawar Suqrat, Corrigan Corinne, White David, Cina Robert A. (2019). Inconsistent and excessive opioid prescribing after common pediatric surgical operations. Journal of Pediatric Surgery.

[ref-51145] Harbaugh Calista M., Gadepalli Samir K. (2019). Pediatric postoperative opioid prescribing and the opioid crisis. Current Opinion in Pediatrics.

[ref-51146] Gaither Julie R., Leventhal John M., Ryan Sheryl A., Camenga Deepa R. (2016). National trends in hospitalizations for opioid poisonings among children and adolescents, 1997 to 2012. JAMA Pediatrics.

[ref-51147] Kaye A.D., Urman R.D., Rappaport Y.. (2019). Multimodal analgesia as an essential part of enhanced recovery protocols in the ambulatory settings. J Anaesthesiol Clin Pharmacol.

[ref-51148] (2018). EXPAREL [Package insert].

[ref-51149] Premier Applied Sciences® (2020). P. I. Premier Healthcare Database White Paper: Data that Informs and Performs. https://products.premierinc.com/downloads/PremierHealthcareDatabaseWhitepaper.pdf.

[ref-51150] Shafi Shahid, Collinsworth Ashley W., Copeland Laurel A., Ogola Gerald O., Qiu Taoran, Kouznetsova Maria, Liao I-Chia, Mears Natalie, Pham An T., Wan George J., Masica Andrew L. (2018). Association of opioid-related adverse drug events with clinical and cost outcomes among surgical patients in a large integrated health care delivery system. JAMA Surgery.

[ref-51151] Rundell Sean D., Resnik Linda, Heagerty Patrick J., Kumar Amit, Jarvik Jeffrey G. (2020). Comparing the performance of comorbidity indices in predicting functional status, health-related quality of life, and total health care use in older adults with back pain. Journal of Orthopaedic & Sports Physical Therapy.

[ref-51152] Chughtai Morad, Sultan Assem A., Hudson Brittany, Goodwin Ryan C., Seif John, Khlopas Anton, Bena James, Jin Yuxuan, Gurd David P., Kuivila Thomas E., Ballock Robert Tracy (2020). Liposomal bupivacaine is both safe and effective in controlling postoperative pain after spinal surgery in children: A controlled cohort study. Clin Spine Surg.

[ref-51153] Dagenais S, Kang A, Scranton R (2016). Impact of bupivacaine liposomal injectable suspension on hospital length of stay and home discharge among Medicare patients undergoing total knee arthroplasty.

[ref-51154] (2020). Adolescent Health.

[ref-51155] Kulak J.A., Griswold K.S. (2019). Adolescent substance use and misuse: Recognition and management. Am Fam Physician.

[ref-51156] Hudgins Joel D., Porter John J., Monuteaux Michael C., Bourgeois Florence T., Alegria Margarita (2019). Prescription opioid use and misuse among adolescents and young adults in the United States: A national survey study. PLOS Medicine.

[ref-51157] Roh Michael S., Kucher Oksana A., Shick Kyle M., Knolhoff Daniel R., McGarvey Jeremy S., Peterson Susan C. (2020). Intramuscular liposomal bupivacaine decreases length of stay and opioid usage following lumbar spinal fusion. Clin Spine Surg.

[ref-51158] Gannon Emmett J, Cornett C.A., Larson E.P., Lyden E.R. (2018). The efficacy of liposomal bupivacaine in lumbar spine surgery. Surgery: Current Trends and Innovations.

[ref-51159] Asche Carl V., Dagenais Simon, Kang Amiee, Ren Jinma, Maurer Brian T. (2019). Impact of treatment with liposomal bupivacaine on hospital costs, length of stay, and discharge status in patients undergoing total knee arthroplasty at high-use institutions. Journal of Medical Economics.

[ref-51160] Cherian J.J., Barrington J., Elmallah R.K., Chughtai M., Mistry J.B., Mont M.A. (2015). Liposomal bupivacaine suspension, can reduce length of stay and improve discharge status of patients undergoing total hip arthroplasty. Surg Technol Int.

[ref-51161] Gan T.J., Oderda G., Robinson S.B. (2012). Opioid-related adverse events increase length of stay and drive up total cost of care in a national database of post surgical patients.

[ref-51162] Aubrun F., Amour J., Rosenthal D., Coriat P., Riou B. (2007). Effects of a loading dose of morphine before i.v. morphine titration for postoperative pain relief: A randomized, double-blind, placebo-control study. British Journal of Anaesthesia.

[ref-51163] Kamerlink Jonathan R, Quirno Martin, Auerbach Joshua D, Milby Andrew H, Windsor Lynne, Dean Laura, Dryer Joseph W, Errico Thomas J, Lonner Baron S (2010). Hospital cost analysis of adolescent idiopathic scoliosis correction surgery in 125 consecutive cases. The Journal of Bone and Joint Surgery-American Volume.

[ref-51164] Oderda Gary M, Evans R.Scott, Lloyd James, Lipman Arthur, Chen Connie, Ashburn Michael, Burke John, Samore Matthew (2003). Cost of opioid-related adverse drug events in surgical patients. Journal of Pain and Symptom Management.

[ref-51165] Brusko G. Damian, Kolcun John Paul G., Heger Julie A., Levi Allan D., Manzano Glen R., Madhavan Karthik, Urakov Timur, Epstein Richard H., Wang Michael Y. (2019). Reductions in length of stay, narcotics use, and pain following implementation of an enhanced recovery after surgery program for 1- to 3-level lumbar fusion surgery. Neurosurg Focus.

