# Supplementary material for: Clinical and Economic Outcomes Associated With Use of Liposomal Bupivacaine Versus Standard of Care for Management of Postsurgical Pain in Pediatric Patients Undergoing Spine Surgery
Source: J Health Econ Outcomes Res. 2021 Apr 14;8(1):29–35. doi: 10.36469/jheor.2021.21967 (PMC8049745; doi:10.36469/jheor.2021.21967)
Supplement: Supplementary Material [file jheor_2021_8_1_21967_57147.pdf]

Journal of Health Economics and Outcomes Research

Supplementary Online Material

Ballock RT, Seif J, Goodwin R, Lin JH, Cirillo J. Clinical and economic outcomes associated with use of liposomal bupivacaine versus standard of care for management of postsurgical pain in pediatric patients undergoing spine surgery. *JHEOR*. 2021;8(1):29-35. doi:10.36469/jheor.2021.21967

Supplemental Table 1. Surgical Procedure Codes
Supplemental Table 2. Definition Codes for Liposomal Bupivacaine Analgesia
Supplemental Table 3. Conversion Factors for In-Hospital Postsurgical Opioid Consumption in Morphine Equivalents
Supplemental Table 4. Unadjusted In-hospital Postsurgical Opioid Consumption Outcome After Primary Spine Surgery in Pediatric Patients
Supplemental Table 5. Unadjusted Opioid-Related Adverse Event Outcomes After Primary Spine Surgery in Pediatric Patients
Supplemental Table 6. Unadjusted Economic Outcomes After Primary Spine Surgery in Pediatric Patients

This supplementary material has been provided by the authors to give readers additional information about their work.

This is an open-access article distributed under the terms of the Creative Commons Attribution 4.0 International License (CCBY-4.0). View this license's legal deed at http://creativecommons.org/licenses/by/4.0 and legal code at http://creativecommons.org/licenses/by/4.0/legalcode for more information.

Supplemental Table 2. Definition Codes for Liposomal Bupivacaine Analgesia

Standard Charge Code	Standard Charge Description	Hospital Charge ID	Hospital Charge Description
250250111850000	BUPIVACAINE, EXPAREL VL 13.3MG/ML (1.3%) 10ML	17540273	65250-0133-09 - BUPIVACAINE LIPOSOME 1.3
250250111850000	BUPIVACAINE, EXPAREL VL 13.3MG/ML (1.3%) 10ML	19957138	65250-0133-09 - BUPIVACAINE LIPOSOME 1.3
250250111850000	BUPIVACAINE, EXPAREL VL 13.3MG/ML (1.3%) 10ML	25083764	EXPAREL 1.3% 13.3MG/ML 10ML
250250111860000	BUPIVACAINE, EXPAREL VL 13.3MG/ML (1.3%) 20ML	21214742	65250-0266-09 - BUPIVACAINE LIPOSOME 1.3
250250120150000	BUPIVACAINE, EXPAREL INJ 1MG	17656090	BUPIVACAINE LIPOSOME(PF) 1.3 % (13.3 MG/M
250250120150000	BUPIVACAINE, EXPAREL INJ 1MG	17676209	BUPIVACAINE LIPOSOME(PF) 1.3 %(13.3 MG/M
250250120150000	BUPIVACAINE, EXPAREL INJ 1MG	18193781	BUPIVACAINE LIPOSOMAL 1.3 % SU
250250120150000	BUPIVACAINE, EXPAREL INJ 1MG	18598752	EXPAREL 1.3 % IJ SUSP
250250120150000	BUPIVACAINE, EXPAREL INJ 1MG	19436080	BUPIVACAINE LIPOSOME(PF) 1.3 % (13.3 MG/M
250250120150000	BUPIVACAINE, EXPAREL INJ 1MG	19672779	BUPIVACAINE LIPOSOMAL 1.3 % SU
250250120150000	BUPIVACAINE, EXPAREL INJ 1MG	20327277	BUPIVACAINE LIPOSOMAL 1.3 % SU
250250120150000	BUPIVACAINE, EXPAREL INJ 1MG	23018001	65250026620 BUPIVACAINE LIPOSOME (PF) 1.3
250250120150000	BUPIVACAINE, EXPAREL INJ 1MG	23794056	HC BUPIVACAINE LIPOSOMAL 1.3 % SUSP 20 M
250250120150000	BUPIVACAINE, EXPAREL INJ 1MG	24246538	HC BUPIVACAINE LIPOSOMAL 1.3 % SUSP 20 M
250250120150000	BUPIVACAINE, EXPAREL INJ 1MG	24438218	BUPIVACAINE LIPOSOME 1.3 % SUSP 20 ML VI
250250120150000	BUPIVACAINE, EXPAREL INJ 1MG	24438366	BUPIVACAINE LIPOSOME 1.3 % IJ SUSP
250250120150000	BUPIVACAINE, EXPAREL INJ 1MG	24500010	BUPIVACAINE LIPOSOME 1.3 % SUSP 20 ML VI
250250120150000	BUPIVACAINE, EXPAREL INJ 1MG	24532715	BUPIVACAINE LIPOSOME 1.3 % SUSP
250250120150000	BUPIVACAINE, EXPAREL INJ 1MG	24588904	BUPIVACAINE LIPOSOME 1.3 % SUSP
250250120150000	BUPIVACAINE, EXPAREL INJ 1MG	24672610	BUPIVACAINE LIPOSOME 1.3 % SUSP
250250120150000	BUPIVACAINE, EXPAREL INJ 1MG	24679655	BUPIVACAINE LIPOSOME 1.3 % SUSP
250250120150000	BUPIVACAINE, EXPAREL INJ 1MG	24687293	BUPIVACAINE LIPOSOME 1.3 % SUSP 20 ML VI
250250120150000	BUPIVACAINE, EXPAREL INJ 1MG	24687573	BUPIVACAINE LIPOSOME 1.3 % SUSP 20 ML VI
250250120150000	BUPIVACAINE, EXPAREL INJ 1MG	24709099	BUPIVACAINE LIPOSOME(PF) 1.3 % (13.3 M
250250120150000	BUPIVACAINE, EXPAREL INJ 1MG	24709674	BUPIVACAINE LIPOSOME 1.3 % SUSP 20 ML VI
250250120150000	BUPIVACAINE, EXPAREL INJ 1MG	24715275	BUPIVACAINE LIPOSOME 1.3 % SUSP 20 ML VI
250250120150000	BUPIVACAINE, EXPAREL INJ 1MG	24720223	BUPIVACAINE LIPOSOME 1.3 % SUSP
250250120150000	BUPIVACAINE, EXPAREL INJ 1MG	24725700	BUPIVACAINE LIPOSOME 1.3 % SUSP 20 ML VI
250250120150000	BUPIVACAINE, EXPAREL INJ 1MG	24731800	BUPIVACAINE LIPOSOME 1.3 % SUSP 20 ML VI
250250120150000	BUPIVACAINE, EXPAREL INJ 1MG	24737277	BUPIVACAINE LIPOSOME 1.3 % SUSP 20 ML VI

Supplemental Table 2. Definition Codes for Liposomal Bupivacaine Analgesia

Standard Charge Code	Standard Charge Description	Hospital Charge ID	Hospital Charge Description
250250120150000	BUPIVACAINE, EXPAREL INJ 1MG	24805351	BUPIVACAINE LIPOSOME 1.3 % SUSP
250250120150000	BUPIVACAINE, EXPAREL INJ 1MG	24834258	BUPIVACAINE LIPOSOME 1.3 % SUSP
250250120150000	BUPIVACAINE, EXPAREL INJ 1MG	24834797	BUPIVACAINE LIPOSOME 1.3 % SUSP
250250120150000	BUPIVACAINE, EXPAREL INJ 1MG	24860599	BUPIVACAINE LIPOSOME 1.3 % SUSP 20 ML VI
250250120150000	BUPIVACAINE, EXPAREL INJ 1MG	24873334	BUPIVACAINE LIPOSOME 1.3 % SUSP
250250120150000	BUPIVACAINE, EXPAREL INJ 1MG	24907306	BUPIVACAINE LIPOSOME 1.3 % SUSP
250250120150000	BUPIVACAINE, EXPAREL INJ 1MG	24910906	BUPIVACAINE LIPOSOME 1.3 % SUSP
250250120150000	BUPIVACAINE, EXPAREL INJ 1MG	24935028	BUPIVACAINE LIPOSOME(PF) 1.3 %(13.3 MG/M
250250120150000	BUPIVACAINE, EXPAREL INJ 1MG	24947622	BUPIVACAINE LIPOSOME 1.3 % IJ SUSP
250250120150000	BUPIVACAINE, EXPAREL INJ 1MG	24964003	65250013309 BUPIVACAINE LIPOSOME (PF) 1.3
250250120150000	BUPIVACAINE, EXPAREL INJ 1MG	24977135	BUPIVACAINE LIPOSOME 1.3 % SUSP 20 ML VI
250250120150000	BUPIVACAINE, EXPAREL INJ 1MG	24989322	BUPIVACAINE LIPOSOME 1.3 % SUSP 20 ML VI
250250120150000	BUPIVACAINE, EXPAREL INJ 1MG	25009443	BUPIVACAINE LIPOSOME 1.3 % SUSP 20 ML VI
250250120150000	BUPIVACAINE, EXPAREL INJ 1MG	25012191	BUPIVACAINE LIPOSOME 1.3 % SUSP 20 ML VI
250250120150000	BUPIVACAINE, EXPAREL INJ 1MG	25018970	BUPIVACAINE LIPOSOME 1.3 % IJ SUSP
250250120150000	BUPIVACAINE, EXPAREL INJ 1MG	25020840	BUPIVACAINE LIPOSOME 1.3 % SUSP 20 ML VI
250250120150000	BUPIVACAINE, EXPAREL INJ 1MG	25033389	BUPIVACAINE LIPOSOME 1.3 % SUSP 20 ML VI
250250120150000	BUPIVACAINE, EXPAREL INJ 1MG	25042118	BUPIVACAINE LIPOSOME 1.3 % SUSP 20 ML VI
250250120150000	BUPIVACAINE, EXPAREL INJ 1MG	25046181	BUPIVACAINE LIPOSOME 1.3 % SUSP 20 ML VI
250250120150000	BUPIVACAINE, EXPAREL INJ 1MG	25062770	BUPIVACAINE LIPOSOME 1.3 % SUSP 20 ML VI
250250120150000	BUPIVACAINE, EXPAREL INJ 1MG	25070458	BUPIVACAINE LIPOSOME 1.3 % SUSP 20 ML VI
250250120150000	BUPIVACAINE, EXPAREL INJ 1MG	25103395	BUPIVACAINE LIPOSOME 1.3 % SUSP 20 ML VI
250250120150000	BUPIVACAINE, EXPAREL INJ 1MG	25104822	BUPIVACAINE LIPOSOME 1.3 % SUSP 20 ML VI
250250120150000	BUPIVACAINE, EXPAREL INJ 1MG	25106164	BUPIVACAINE LIPOSOME 1.3 % SUSP 20 ML VI
250250120150000	BUPIVACAINE, EXPAREL INJ 1MG	25115374	BUPIVACAINE LIPOSOME 1.3 % SUSP
250250120150000	BUPIVACAINE, EXPAREL INJ 1MG	25123633	INJECTION EXPAREL MG
250250120150000	BUPIVACAINE, EXPAREL INJ 1MG	25139931	BUPIVACAINE LIPOSOME 1.3 % SUSP 20 ML VI
250250120150000	BUPIVACAINE, EXPAREL INJ 1MG	25156832	BUPIVACAINE LIPOSOME 1.3 % SUSP 20 ML VI
250250120150000	BUPIVACAINE, EXPAREL INJ 1MG	25172121	BUPIVACAINE LIPOSOME 1.3 % SUSP 20 ML VI
250250120150000	BUPIVACAINE, EXPAREL INJ 1MG	25179604	BUPIVACAINE LIPOSOME 1.3 % SUSP 20 ML VI

Supplemental Table 2. Definition Codes for Liposomal Bupivacaine Analgesia

Standard Charge Code	Standard Charge Description	Hospital Charge ID	Hospital Charge Description
250250120150000	BUPIVACAINE, EXPAREL INJ 1MG	25184235	BUPIVACAINE LIPOSOME 1.3 % SUSP
250250120150000	BUPIVACAINE, EXPAREL INJ 1MG	25185832	BUPIVACAINE LIPOSOME 1.3 % SUSP 20 ML VI
250250120150000	BUPIVACAINE, EXPAREL INJ 1MG	25187213	BUPIVACAINE LIPOSOME 1.3 % SUSP 20 ML VI
250250120150000	BUPIVACAINE, EXPAREL INJ 1MG	25199510	BUPIVACAINE LIPOSOME 1.3 % SUSP 20 ML VI
250250120150000	BUPIVACAINE, EXPAREL INJ 1MG	25205840	BUPIVACAINE LIPOSOME 1.3 % SUSP 20 ML VI
250250120150000	BUPIVACAINE, EXPAREL INJ 1MG	25214594	BUPIVACAINE LIPOSOME 1.3 % SUSP 20 ML VI
250250120150000	BUPIVACAINE, EXPAREL INJ 1MG	25315366	BUPIVACAINE LIPOSOME 1.3 % SUSP
250250120150000	BUPIVACAINE, EXPAREL INJ 1MG	25333250	BUPIVACAINE LIPOSOME 1.3 % SUSP
250250120150000	BUPIVACAINE, EXPAREL INJ 1MG	25343295	BUPIVACAINE LIPOSOME 1.3 % SUSP
250250120150000	BUPIVACAINE, EXPAREL INJ 1MG	25345126	BUPIVACAINE LIPOSOME 1.3 % SUSP
250888002710000	BUPIVACAINE PARENTERAL MISC	22499392	65250-0266-09 - BUPIVACAINE LIPOSOME 1.3

Supplemental Table 3. Conversion Factors for In-Hospital Postsurgical Opioid Consumption in Morphine Equivalents

	Route	Conversion (Multiplication) Factor	
		**PO**	**IV**
Fentanyl	IV, IM, IT, PO	300	100
Hydromorphone	IV	20	6.67
Hydromorphone	PO	4	1.3
Meperidine/Pethidine	IV, SC	0.3	0.1
Meperidine/Pethidine	PO	0.1	0.033
Morphine	IT	300	100
Morphine	IV, IM, SC	3	1
Morphine	PO	1	0.33

Abbreviations: IM, intramuscular; IT, intrathecal; IV, intravenous; PO, oral; SC, subcutaneous.

Supplemental Table 4. Unadjusted In-hospital Postsurgical Opioid Consumption Outcome After Primary Spine Surgery in Pediatric Patients

	LB	Non-LB
In-hospital postsurgical opioid consumption, MMEs	1337	2659

Abbreviations: LB, liposomal bupivacaine; MME, morphine milligram equivalent.

Supplemental Table 5. Unadjusted Opioid-Related Adverse Event Outcomes After Primary Spine Surgery in Pediatric Patients

	LB	Non-LB
Patients with ORAEs, %	22.52	21.94

Abbreviations: LB, liposomal bupivacaine; ORAE, opioid-related adverse event.

Supplemental Table 6. Unadjusted Economic Outcomes After Primary Spine Surgery in Pediatric Patients

	LB	Non-LB
LOS, days	4.3	5.1
Total hospital care costs, US $	50 917	41 595
Major cost items (%), US $		
Room and board	9420 (18)	9266 (22)
Central supply	28 410 (56)	18 729 (45)
Pharmacy	1324 (3)	1810 (4)
Surgery	7972 (16)	6975 (17)

Abbreviations: LB, liposomal bupivacaine; LOS, length of stay.
